# Guidelines for preventing and slowing myopia progression in Brazilian children

**DOI:** 10.5935/0004-2749.2023-0009

**Published:** 2024-07-10

**Authors:** Fábio Ejzenbaum, Tania Mara Cunha Schaefer, Celso Cunha, Julia Dutra Rossetto, Izabela F. Godinho, Célia Regina Nakanami, Regina K. Noma, Luisa Moreira Hopker

**Affiliations:** 1 Department of Ophthalmology, Santa Casa de Misericórdia de São Paulo, São Paulo, SP, Brazil; 2 Clínica Schaefer Research Center, Curitiba, PR, Brazil; 3 Hospital de Olhos de Cuiabá, Cuiabá, MT, Brazil; 4 Pediatric Ophthalmology Department, Instituto de Puericultura e Pediatria Martagão Gesteira, Universidade Federal do Rio de Janeiro, Rio de Janeiro, RJ, Brazil; 5 Departmernt of Ophthalmology, Hospital Evangélico, Belo Horizonte, MG, Brazil; 6 Ophthalmology and Visual Sciences Department, Escola Paulista de Medicina, Universidade Federal de São Paulo, São Paulo, SP, Brazil; 7 Ophthalmology, Hospital das Clínicas, Faculdade de Medicina, Universidade de São Paulo, São Paulo, SP, Brazil; 8 Department of Ophthalmology, Hospital de Olhos do Paraná, Curitiba, PR, Brazil

**Keywords:** Myopia, Pupil disorders, Disease progression, Atropine, Refraction, Ocular, Mydriatics, Contact lens, Biometry, Child, Brazil, Miopia, Distúrbios pupilares, Progressão da doença, Atropina, Refração ocular, Midriáticos, Lentes de contato, Biometria, Criança, Brasil

## Abstract

This document on myopia control is derived from a compilation of medical literature and the collective clinical expertise of an expert committee comprising members from the Brazilian Society of Pediatric Ophthalmology and the Brazilian Society of Contact Lenses and Cornea. To manage myopia in children, the committee recommends corneal topography and biannual visits with cycloplegic refraction, along with annual optical biometry. For fast-progressing myopia, biannual biometry should be considered. Myopic progression is defined as an annual increase in spherical equivalent greater than 0.50 D/year or in axial length greater than 0.3 mm (until 10 years old) or 0.2 mm (above 11 years). The proposed treatments for myopia progression include environmental control, low concentration atropine, defocus glasses, contact lenses, or Ortho-K lenses, and combinations of these methods may be necessary for uncontrolled cases. Treatment should be sustained for at least 2 years. This document serves as a comprehensive guideline for diagnosing, treating, and monitoring pre-myopic and myopic children in Brazil.

## Abstract (pt)

Esta revisão foi baseada na literatura médica e na experiência clínica de um comitê de especialistas membros da Sociedade Brasileira de Oftalmologia Pediátrica e da Sociedade Brasileira de Lentes de Contato e Córnea. Rotineiramente as crianças devem ser submetidas a topografia da córnea no primeiro exame e visitas semestrais com refração cicloplegiada e biometria óptica anual. A progressão da miopia foi definida como um aumento anual no equivalente esférico maior que 0,50 D/ano ou do comprimento axial maior que 0,3 mm (até 10 anos) ou 0,2 mm (mais de 11 anos). Os tratamentos propostos para a progressão são controle ambiental, atropina em baixa concentração, óculos com defocus, lentes de contato ou ortoceratologia, devendo-se considerar associações para casos não controlados. O tratamento deve ser realizado por pelo menos 2 anos. O presente documento é uma diretriz para diagnóstico, tratamento e acompanhamento de crianças pré-míopes e míopes no Brasil.

## INTRODUCTION

Uncorrected myopia represents a significant cause of preventable visual impairment, having potential impacts on children’s learning, school performance, and self-esteem^(1,2)^. Studies conducted on samples of campaigns and collective efforts among school-age children in Brazil have indicated an overall prevalence ranging from 2.1% to 13.3%. Despite an increasing prevalence, both in Brazil and other South American countries, it appears that the myopic shift and myopia progression are comparatively lower than in other regions worldwide^(3,4)^. Furthermore, meta-analyses and population-based studies have revealed temporal trends showing an increased prevalence among children, particularly with a higher likelihood in girls compared to boys. The onset of myopia has been associated with parental myopia, and there has been a notable increase among adolescents, along with a higher prevalence in urban areas compared to rural areas in Asia^(5-7)^.

The onset of myopia during the early school years (ages 5-7) is associated with faster progression and higher rates of myopia and eye growth throughout childhood and adolescence, especially among individuals exposed to environmental and behavioral factors related to myopia progression^(1,5,8)^. The age of myopia onset and/or the duration of its progression are crucial predictors of high myopia in late childhood or adolescence^(9)^. Children experiencing early onset and rapidly advancing progression are prone to developing high myopia (≤-6 diopters (D)), leading to potential complications in adulthood that may result in irreversible visual impairment^(10,11)^.

Among the main complications associated with high myopia are myopic macular degeneration (affecting 1%-4% of the general population in some countries), cataract, glaucoma, retinal detachment, and optic neuropathy^(12-13)^. Several environmental factors contribute to the changes in lifestyle that increase the risk of myopia progression. These factors include spending less time outdoors, high-pressure educational systems (especially in early ages in East Asian countries), continuous and excessive use of electronic devices (mainly tablets and cell phones) and engaging in other close-up activities^(1,8)^. The objective of this study is to provide guidance on the evaluation and management of myopia progression in children.

## METHODS

This guideline was developed by reviewing the existing literature and drawing upon the clinical expertise of experts from the Brazilian Society of Pediatric Ophthalmology (SBOP) and the Brazilian Society of Contact Lenses and Cornea (SOBLEC). The review encompassed the prevalence, classification, progression, and treatment of myopia, with data gathered from PubMed up to April 2023. The search utilized various terms such as myopia AND children OR classification OR prevalence OR progression OR treatment (progression AND myopia AND treatment). The group selected and analyzed 83 scientific papers, comprising meta-analyses, systematic reviews, randomized controlled trials (RCTs), case-control studies, observational studies, and case reports. To assess the quality of evidence, the studies were classified based on Guyatt et al.’s^(14)^ criteria: Level I involved two or more high-quality RCTs, studies with high evidence level by the Grading of Recommendations Assessment, Development, and Evaluation (GRADE), or statements from other guidelines with level A of evidence (experimental or observational studies with higher consistency). Level II evidence was established when there were a limited number of RCTs, multiple controlled but non-randomized studies, or several RCTs of lower quality. Additionally, evidence at this level could be derived from cohort or case-control studies, preferably conducted by multiple research groups or from multiple centers. Furthermore, clear-cut effects observed in non-controlled studies, studies with moderate evidence level according to GRADE, or statements from other guidelines with level B of evidence (experimental or observational studies with lower consistency) were also considered as Level II^(14-15)^. On the other hand, Level III evidence relied on expert opinions, clinical experiences, descriptive studies^(15-17)^, cohort or case-control studies of lower quality, studies with low or very low evidence level by GRADE, or statements from other guideline with level C or D of evidence (case reports studies or specialist opinion-based consensus)^(14-17)^.

The final guideline document received approval from all the representatives of the involved societies. Since there was no involvement of human subjects, ethics approval was not required and was therefore waived.

## RESULTS

The guidelines presented here for monitoring and treating myopia progression in children are based on a thorough review of the current literature and the clinical expertise of the expert group. This document encompasses the latest concepts on myopia classification, risk factors associated with myopia progression, recommended ophthalmologic visits and ancillary examination regimens, as well as strategies for preventing and treating myopia progression.

### Myopia classification

Myopia is defined quantitatively as a condition where the spherical equivalent refractive error (SER), with relaxed ocular accommodation, is ≤-0.5 D. According to IMI, low myopia is characterized by a refractive error between ≤-0.5 and >-6.00 D, whereas high myopia is when the refractive error is ≤-6.00 D^(17)^. However, it is important to note that the World Health Organization (WHO) classifies high myopia as a refractive error above -5.00 D^(21,22)^.

Myopia can be further categorized into refractive and axial types. In refractive myopia, the optical power of the cornea and/or crystalline is high in eyes with a normal axial length (AL). Conversely, in axial myopia, the optical axis is too long compared to the refractive power of the cornea and lens. Axial and refractive myopia are often considered as distinct entities.

A definition for pre-myopia is also worth noting. It includes children with refractive error status of <+0.75 D at 6 years old, ≤+0.50 D between 7 and 8 years, ≤+0.25 D at 9-10 years, and ≤0 D at 11 years^(16-18,21)^. Pre-myopia is of significance as it identifies children who are at a high risk of developing myopia in the future.

### Risk factors for fast-progressing myopia

Based on current literature, myopia tends to progress faster in younger children and decelerates with age^(1,22)^. Early onset of myopia or a prolonged duration of myopia progression are the most significant predictors of high myopia^(2,5,23)^ (Level I). Heredity also plays a crucial role in myopia development, with approximately 150 genetic loci identified as contributing to the condition^(24)^. Additionally, studies on parental myopia have revealed that having one myopic parent triples the risk of myopia, while having both parents with myopia increases the risk sevenfold^(25,26)^. Various factors influence myopia progression, including ethnicity (more common among Asians), parents with a higher education level, less time spent outdoors, and engagement in schooling/near work activities^(1)^.

A rapid myopia progression is considered to occur when there is an increase in refractive error increase rate of 0.75 D/year or higher^(3,26)^. Risk factors for fast-progressing myopia include the following:

- Age younger than 7 years old^(26)^ (Level I)
- Ethnicity (Asian)^(26)^ (Level II)
- Parental myopia^(26)^ (Level II)
- Limited time spent outdoors^(27)^ (Level I)
- Prolonged durations of near work/screen time (>45 minutes), continuous use, and very close use (<25 cm)^(28)^ (Level II)

### Ophthalmologic evaluation

Myopia management requires adherence to a clinical protocol for closely monitoring the progression of the SER and AL. When myopic progression is suspected, an ophthalmologic evaluation should be conducted every 6 months, including medical history and the following components^(18)^:

- Clinical history: Assessing visual impairment for distance, if applicable, age since commencement of wearing glasses, family history of myopia, and lifestyle risk factors (e.g., parents’ education level, outdoor time, near work-digital screen time, etc.)
- Examinations:

Best-corrected visual acuity for distance and near vision

Evaluation of accommodative and binocular vision

Anterior biomicroscopy

Cycloplegic refraction-utilizing the recommended cycloplegia protocol: one drop of 0.5% proxymetacaine, followed by one drop of 1% cyclopentolate, and then one drop of 1% tropicamide given 0-5 minutes apart. The test should be performed 30-40 minutes after the first drop^(29)^.

### Retinal imaging

Additional exams (if available):

- Annual measurement of AL (biannually in fast-progressing cases) using non-contact devices like Optical Biometry
- Corneal topography (to exclude keratoconus or to determine the requirement for contact lens fitting)

Myopic progression diagnosis and indication for treatment:

- Cycloplegic refractive error examination: an increase higher than SER -0.50 D over 1 year (with exceptions considered (≥0.5) for cases of early-onset myopia (<7 years) and with other fast-progressing risk factors)^(30)^
- AL: an axial growth of 0.3 mm/year (until 10 years old) or 0.2 mm/year (above 10 years old)

NOTE: Emmetropes typically have an AL of 22-24.5 mm, while axial lengths greater than 25 mm are generally associated with myopia^(18)^.

### Myopia prevention

Children with fast progression risk factors and those identified as pre-myopes should be carefully monitored to prevent or delay the early onset or progression of myopia. One RCT showed that increased time spent outdoors may offer some protection against myopia onset^(30)^. Moreover, it can slow down myopia progression, although the effect might not be clinically significant. Therefore, encouraging outdoor activities should be viewed as an additional treatment to complement other interventions for myopia control (Level I), rather than a standalone solution^(30,31)^. A population-based article conducted in Taiwan, which analyzed the myopic shift in preschool children (ages 5-6) encouraged to engage in outdoor activities (at least 30 minutes daily), reported a nearly 50% reduction in the incidence of myopia^(32)^. It appears that increased outdoor exposure can reduce the risk of myopia onset, but it may not have a significant impact on myopic progression in individuals already diagnosed with the condition.

### Treatment for reducing myopia progression

#### Atropine for myopic control

Atropine is a muscarinic drug, but its exact site of action remains undetermined. Different concentrations of atropine have been found to be effective in controlling myopia: low concentrations (0.01%, 0,025%, or 0.05%), medium concentrations (0.075%-0.1%), and high concentrations (above 0.1%)^(33,34)^ (Level I). Control with low concentrations has been observed to be dose-dependent (Level I)^(35)^, while higher concentrations have shown better myopia control (Level I)^(36)^. However, the use of high concentrations can lead to more significant side effects, such as reduced amplitude of accommodation, glare, photophobia, and headaches^(37)^.

Studies have indicated that low concentrations of atropine can control approximately 40%-70% of myopia progression in the Asian population^(38-39)^ (Level I). The most effective long-term dose (balancing control, side effects, and rebound) has not yet been fully established. After discontinuing atropine treatment, some patients may experience a rebound effect (Level I)^(36)^; therefore, the optimal duration of treatment remains uncertain. The current treatment involves administering one drop of atropine at night, to be continued for a minimum of 2 years, but it may extend until the child reaches 15-16 years old (Level I)^(33,36-38)^. To avoid potential rebound effects, some researchers propose a gradual tapering of the atropine concentration instead of abrupt stop, although detailed studies on this approach are still lacking^(30)^. The reduction can be accomplished by gradually reducing the concentration for at least 2 months, and once it reaches 0.01%, it can be used on alternate days for a brief period before discontinuation.

Using atropine at a low concentration in children aged 5 years and older is considered safe, whereas younger children may require a higher dose for effective myopia control (e.g., 0.05%)^(34)^ (Level I). Adverse events related to atropine use are rare and may include allergies, mydriasis, and reduced accommodation. Therefore, discussing the use of atropine with the child’s family is essential. Atropine is not recommended for patients with astigmatism exceeding 1.50 or 2.50, corneal ectasia, neurological diseases, or those with a known allergy to atropine^(37)^. Its use in individuals with Down Syndrome and high myopia lacks sufficient support from published studies; hence, predicting the expected outcomes or the safety of the atropine treatment in these cases is not possible^(35,38)^.

According to this expert consensus, when treating with atropine for myopia control, it is recommended to follow a biannual follow-up schedule for monitoring refractive status (Level IIID) and annual follow-up for biometry (Level I). Based on the previous literature review (all Level I evidence) and clinical experience, several possibilities for atropine prescription for myopia control are presented below:

1. Begin with low-dose atropine (0.01%) for 1 year. If the desired control is not achieved, increase the dose to 0.025%. If still uncontrolled in the subsequent year, further increase the dose to 0.05%^(30)^ (Level I).
2. Start the dose between 0.01% and 0.025% based on risk evolution criteria, such as child’s young age, parental history of myopia, myopia progression rate (MPR), refraction, and axial diameter.
3. For children aged between 5 and 8 years: Initiate treatment with 0.025% or 0.05% atropine. For children aged between 9 and 15 years:
a. If MPR is 0.50D/year or refraction is less than or equal to 4 D, and/or AXL is less than 24.5 mm, start with 0.01% atropine.
b. If MPR is higher than 0.50 D/year or refraction over -4 D, and/or AXL is larger than 24.5 mm, start with 0.025% atropine.

For patients aged over 15 years: Initiate treatment with 0.01% atropine (Level I)^(33,35,36,39)^.

### Hyperopic defocus for myopia treatment

Peripheral hyperopic defocus (PHD) refers to an opti-cal abnormality where hypermetropia (farsightedness) induced in the midperiphery of the retina causes a phenomenon of defocusing. This occurs due to the conical shape of the eye, leading to hypermetropic blur in the peripheral retina. The peripheral retina is believed to play a role in controlling AL growth, and the presence of PHD has been recognized as a potential risk factor for myopia progression in humans^(40-42)^. Hung et al.^(43)^ (Level III) proposed that an increase in the area of PHD can result in a reduction of neuromodulators, such as dopamine, being released in the peripheral retina. This increase in the area of PHD is linked to the weakening of the structural integrity of the sclera, which consequently leads to an increase in AL.

### Lens wear in the hypermetropic defocus treatment

Based on the peripheral defocus theory, new custom-designed lenses aim to minimize PHD. Several recent ophthalmic lens designs for myopia control are as follows:

1. *Defocus Incorporated Multiple Segments (DIMS)/Mul-tisegment of Myopic Defocus Spectacle Lens* (developed by Hoya)^(44)^. In a randomized study, it was found that myopia progression was significantly reduced by 59% and AL elongation decreased by 60% when compared to wearing single-vision lenses (Level I)^(45)^. Another 2-year randomized clinical trial involved children aged 8-13 years (n=160) with myopia from -1.00 to -5.00 D and astigmatism up to 1.50 D. The trial compared children using single-vision glasses with those using DIMS lenses. The results showed that children wearing DIMS lenses had 52% less myopia progression and 62% less axial elongation compared to the control group. Additionally, 21.5% of DIMS lens wearers experienced no myopia progression during the study period, in contrast to 7% of those wearing single-vision glasses (Level I)^(44)^. After a 3-year period, 120 children who initially wore single-vision glasses switched to using DIMS lenses. The positive results in myopia control were sustained over an additional 3 years (n=65). Even those children who underwent DIMS lens replacement (n=55) responded well to the treatment, as evident from an improved progression curve (Level I)^(45)^. In a recent study with a 6-year follow-up, the paper evaluated four groups: Group 1 (n=36) wore DIMS spectacles for 6 years; Group 2 (n=14) wore DIMS lens for the first 3.5 years and switched to SV spectacles afterward; Group 3 (n=22) used SV spectacles for the first 2 years and then switched to DIMS lenses; Group 4 (n=18) wore SV spectacles for the first 2 years, then switched to DIMS lenses for 1.5 years, and finally returned to SV spectacles again. The study findings revealed that Group 1 showed no significant differences in myopia progression (-0.52 ± 0.66 vs. -0.40 ± 0.72 D) and axial elongation (0.32 ± 0.26 vs. 0.28 ± 0.28 mm, both p>0.05) between the first and subsequent 3 years. In the last 2.5 years, the groups using DIMS lenses (Groups 1 and 3) displayed less myopia progression and axial elongation than the groups using single-vision lenses (Groups 2 and 4)^(46)^ (Level I).
2. *Highly Aspherical Lenslet Target* or *HALT Technology* (developed by Essilor)^(47)^. This technology demonstrated a remarkable 67% deceleration in myopia progression, on average, compared to single-vision lenses worn for 12 hours. A two-year randomized study with 157 children aged 8-13 years showed a significant reduction in myopia progression for both SE and AL by 67% (0.99 D) and 60% (0.41 mm), respectively, when compared to single-vision lenses (Level I)^(48)^.

Lenses equipped with DIMS and HALT technology share the following characteristics:

• A single central vision zone for distance
• A single-vision correction in the peripheral zone
• An intermediate zone (treatment area) containing mul-tiple segments (DIMS) or lenslets (HALT) designed to create a differential myopic blur in front of the retina, with spaces between them for single-vision correction
• No alteration in binocular vision or accommodation
• A strict requirement for proper centralization of the lens on the central clear zone to ensure better acuity and defocus treatment in the midperiphery, as well as no serious vision complaints for distance and middle periphery

3) Perifocal defocus spectacle lens (developed by IOT/Art Optica). This lens creates a positive asymmetric defocus (largest in the temporal area) on the horizontal meridian. In a study involving a Caucasian population, after 5 years, myopia progression was sig-nificantly reduced from -1.95 ± 0.2 D in the control group versus -1.16 ± 0.2 D in the treated group, with a difference of 0.79 D between them. The AL elongation was decreased by 56% (0.46 mm ± 0.05 vs 0.71 ± 0.09 mm) in 2 years when compared to wearing single-vision lenses^(49)^. (Level I).

### Defocus contact lenses in myopia control

Myopia control studies with multifocal contact lenses have yielded varying results, with reductions in myopia progression rates (SE) ranging from 0 to 72% to 80%^(50-52)^.

One notable study called BLINK^(53)^ (Bifocal Lenses in Nearsighted Kids) compared multifocal CL with simple vision CL in 292 children aged between 7 and 11 years. The study, which had a follow-up period of 3 years, demonstrated a reduction in myopia progression by 43% and AL by 36% (Level I). The efficacy of the treatment was more significant in the higher addition group (+2.50 D addition), which could be particularly relevant for younger children who have positive individual risk factors for myopia progression.

The CooperVision MiSight lens is currently the only disposable lens approved in Brazil for myopia control. A 7-year clinical study involving the MiSight lens showed no apparent rebound effect (Level I)^(54)^. In a multicenter, prospective, randomized, double-blind study, MiSight lenses were compared with single-vision spherical disposable soft lenses in children aged 8-12 years with myopia ranging from -0.75 to -4.00 D and astigmatism <1.00 D. The study revealed reductions in myopia progression (59%) and AL (52%) during a 3-year follow-up period (Level I)^(54)^. After 6 years of follow-up, 23% of the patients experienced a refractive change of less than 0.25 D (spherical equivalent). Even the original control group, which switched to MiSight lenses in the fourth year of follow-up also, exhibited a reduction in myopia progression and AL growth (0.81 mm) over the subsequent 3 years.

### ORTHO-K in myopia control

Orthokeratology is a technique used for temporary reduction of refractive errors. It involves using specially designed reverse curve contact lenses that apply positive pressure to the center of the cornea, aiming to reshape it. The lens effects are reversible and can either disappear within a few hours or last for a day or longer. This treatment has no age restrictions and can be used for individuals of all ages. It works by reducing the thickness of the central epithelium of the cornea through the redistribution of intracellular fluid from these cells to the intracellular space of the midperipheral epithelial cells. The contact lens exerts negative pressure at the midperipheral region, leading to the thickening of the corneal epithelium in that area. It is important to note that no cellular displacement occurs during this process; instead, there is a redistribution of intracellular fluid within the corneal epithelium^(55)^.

Corneal remodeling is responsible for a temporary reduction in the AL of the eye, effectively correcting myopia. According to findings in published literature, this corneal remodeling also contributes to reducing myopia progression in 35%-60% of patients. It achieves by forming an elevation in the corneal midperiphery, which results from thickening induced by the migration of intracellular fluid. This elevation leads to a refractive alteration that corrects the hypermetropic defocus in the midperiphery of the retina (Level I and II)^(54-61)^. For routine evaluation, in addition to the standard examinations used for myopic children evaluation, computerized topography with axial and tangential maps, as well as specular corneal microscopy and tomography (Galilei or Pentacam), are recommended as complementary examinations.

Patients with regular corneas are generally more suitable for orthokeratology treatment. Existing literature indicates that the adverse effects of orthokeratology treatment are reversible and comparable to those seen with other types of contact lenses designed for night wear^(62-68)^. However, there are some contraindications to consider:

- Clinical contraindications: Patients with inflammation or infections in the anterior segment of the eye (bacterial, viral, or fungal); abnormalities in the conjunctiva, cornea, or eyelid; corneal hypoesthesia; dry eye; systemic conditions that affect the eyes, the lacrimal pathways, and tearing should not undergo orthokeratology treatment.
- Corneal contraindications: Patients with against-the-rule astigmatism, corneal ectasia, astigmatism exceeding half the spherical degree, cylinders larger than 1.75 D, and corneas flatter than 40 D or more curved than 48 D and those who, after treatment, show a corneal curvature below 38 D should not be considered for orthokeratology treatment.

### Combined treatments

Few studies have explored the association of treatments, although it is possible that a combined effect exists when treatments with different mechanisms of action are used together. Some studies have suggested that combining atropine and Ortho-K treatment may lead to a greater effect in slowing axial elongation in children with myopia compared to Ortho-K monotherapy (Level II)^(69)^. A recent meta-analysis comparing Ortho-K to Ortho-K plus atropine found four eligible studies with a total of 267 subjects (Level I)^(70)^. The analysis revealed that the mean AL in the experimental group was 0.09 mm shorter than in the control group (WMD=-0.09, 95%CI [-0.15, -0.03], p=0.003). However, no significant differences were observed between the two groups in terms of uncorrected distant visual acuity, corneal endothelial cell density, or intraocular pressure (WMD was -0.01 [95% CI: -0.03, 0.01], 11.75 [95% CI: -4.09, 27.58], 0.12 [95% CI: -0.40, 0.63], respectively). None of the studies reported severe adverse events. Kinoshita et al. reported a better combined treatment effect, especially in the first year^(69)^ (0.09 mm vs. 0.19 mm), but the difference between the two treatments diminished in the second year (0.20 mm vs. 0.21 mm).

The combination treatment showed a more pronounced additive effect in slowing axial growth among children with lower myopia, whereas monotherapy was equally effective as the combination in children with higher myopia. The key factor lies in the myopia correction achieved with Ortho-K therapy. In children with higher myopia, Ortho-K provides a larger myopia correction, resulting in improved defocus on the peripheral retina. Conversely, in children with lower myopia, the extent of myopia correction with Ortho-K is smaller, and this might not sufficiently improve the defocus on the peripheral retina through monotherapy alone. In such cases, adding 0.01% atropine seems to be more effective. The study suggests that combining Ortho-K and 0.01% atropine yields greater effectiveness in slowing axial elongation in children with myopia, particularly in cases with a relatively short duration of treatment.

In a recent study, conducted in a European population with 146 participants, the participants were divided into 4 groups: 53 received 0.01% atropine, 30 used DIMS spectacles, 31 received a combination of 0.01% atropine and DIMS, and 32 used the single-vision control spectacles. After 1 year of treatment, the group receiving the atropine and DIMS showed a significant reduction in myopia progression compared to both the DIMS-only and atropine-only groups. Interestingly, during this period, there was no difference in myopia progression between the atropine and DIMS groups^(71)^ (Level II).

## DISCUSSION

The strategies described here play a crucial role in managing myopia progression and are essential to minimize the number of high myopes and their possible consequences. The choice of treatment should be informed by the expertise of the healthcare professional and the socioeconomic context. It is important to acknowledge that in countries like Brazil, with limited resources and as a low-income country, access to therapies such as glasses or contact lenses may not be available for most of the population. Thus, this factor needs to be carefully considered before making the initial prescription.

### Suggested treatment strategies (Flowchart)

**Figure 1:**
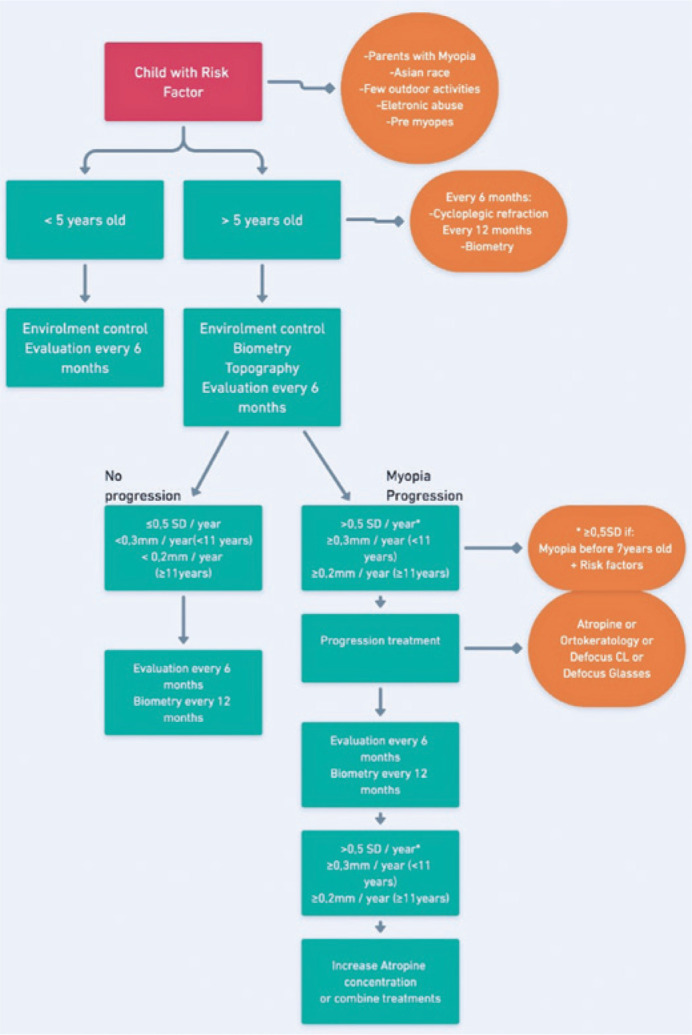
Treatment and control flowchart of myopic children.

Based on the current scientific evidence and the consensus of the professionals involved in developing the guidelines and the Brazilian reality, a flowchart has been created for the follow-up and control of myopic children (Figure 1).

When choosing the appropriate method, healthcare professionals should take into account various factors such as cost, astigmatism (defocus CL correcting up to 0.75 DC), ophthalmologist’s personal experience, and the perception of invasiveness associated with a particular treatment.

For cases where monotherapy proves insufficient in controlling myopia progression, treatment options may involve increasing atropine concentration (in cases where the drug is already being used) or combining it with another therapeutic approach. In such situations, the decision should consider the healthcare professional’s personal experience or familiarity with the selected method.

The treatment duration is determined based on the refractive error stability and the AL (Figure 1).
